# Fibre Type–Specific Proteomics Reveals Shared and Distinct Skeletal Muscle Adaptations to Resistance Training and Beta_2_‐Adrenergic Agonist

**DOI:** 10.1002/jcsm.70175

**Published:** 2026-01-25

**Authors:** Søren Jessen, Andrea Di Credico, Roger Moreno‐Justicia, Lukas Moesgaard, Anders Lemminger, Ben Stocks, Angela Di Baldassarre, Jens Bangsbo, Atul S. Deshmukh, Morten Hostrup

**Affiliations:** ^1^ Clinical and Experimental Physiology University of Copenhagen Copenhagen Denmark; ^2^ The August Krogh Section for Human and Molecular Physiology, Department of Nutrition, Exercise and Sports University of Copenhagen Copenhagen Denmark; ^3^ Department of Medicine and Aging Sciences University “G. d'Annunzio” of Chieti—Pescara Chieti Italy; ^4^ The Novo Nordisk Foundation Center for Basic Metabolic Research, Faculty of Health and Medical Sciences University of Copenhagen Copenhagen Denmark

**Keywords:** beta_2_‐agonist, exercise mimetic, muscle growth, salbutamol

## Abstract

**Background:**

Skeletal muscle is essential for metabolic health and physical function. While resistance training promotes muscle hypertrophy, alternative therapeutic strategies are needed for individuals unable to engage in physical activity. Because beta_2_‐adrenergic stimulation induces muscle growth without mechanical load, we assessed muscle fibre type–specific proteomic adaptations to prolonged beta_2_‐adrenergic stimulation and resistance training to decipher shared and distinct remodelling patterns.

**Methods:**

We collected vastus lateralis biopsies from 21 moderately trained young males (mean ± SD, age: 24 ± 3) before and after 4‐week whole‐body resistance training (three sessions/week) or daily inhalation of beta_2_‐adrenergic agonist terbutaline (4 mg/day). From each biopsy, we isolated 40 muscle fibres and typified them using myosin‐heavy‐chain markers. Fibre pools were analysed using LC–MS/MS‐based proteomics.

**Results:**

Beta_2_‐adrenergic stimulation and resistance training both increased peak‐power output during bike‐ergometer sprinting (+36 W; 95% CI: 11 to 61, *p* = 0.007 and +27 W; 95% CI: −1 to 56, *p* = 0.062, respectively) with no between‐treatments differences (treatment × time interaction: *p* = 0.644). Beta_2_‐adrenergic stimulation regulated 15 and 23 proteins in Type I and Type II fibres, respectively, compared to 101 and 65 with resistance training. There was a remarkable fibre type–dependent response, with ~7% of regulated proteins shared between Type I and Type II fibres with resistance training and ~3% with beta_2_‐adrenergic stimulation. Both interventions increased abundance of ribosomal proteins, in which resistance training induced a 25% increase in Type I fibres (*p* < 0.001) but only 3% in Type II (*p* = 0.374), while beta_2_‐adrenergic stimulation increased ribosomal proteins in both fibre types (Type I: 6% increase, *p* = 0.008; Type II: 9% increase, *p* < 0.001). Mitochondrial electron‐transport‐chain protein abundances decreased with both interventions: resistance training reduced abundances mainly in Type I fibres (17% decrease, *p* < 0.001; Type II: 5% decrease, *p* = 0.147), while beta_2_‐adrenergic stimulation caused uniform decreases (Type I: 7% decrease, *p* = 0.018; Type II: 9% decrease, *p* = 0.001). Resistance training uniquely increased contractile, cytoskeletal and extracellular matrix proteins, which was not mimicked by beta_2_‐adrenergic stimulation. S100A13 was upregulated across both interventions and fibre types, whereas MUSTN1 was regulated exclusively with resistance training. Knock‐down of S100a13 (−52%; *p* < 0.001) and Mustn1 (−96%; *p* < 0.001) in C2C12 myotubes impaired myotube formation (fusion index: S100a13: −5%; *p* = 0.002; Mustn1: −21%; *p* < 0.001).

**Conclusions:**

Beta_2_‐adrenergic stimulation induces proteomic adaptations that partially mimic resistance training, particularly in ribosomal proteins. Shared regulation of S100A13 and unique regulation of MUSTN1 with resistance training suggest distinct and complementary roles in regulating muscle growth. These findings indicate that the beta_2_‐adrenergic receptor is a potential target to counter muscle atrophic conditions, offering a pharmacological approach for individuals unable to engage in resistance training.

## 1 Introduction

Skeletal muscle plays a critical role for metabolic regulation and function, making it a prime target for therapeutic intervention in pathological conditions such as muscle atrophy or insulin resistance. While resistance training is first line therapy to promote muscle growth and improve its function, many individuals are unable to engage in physical activity. This has sparked interest in developing alternative therapies that mimic the response to resistance exercise intrinsically within individual muscle fibres but without imposing mechanical load. The beta_2_‐adrenergic receptor is one such target.

Research across multiple disciplines demonstrates that the beta_2_‐adrenergic receptor is highly important for muscle mass and function. It constitutes >90% of the adrenoceptor population in skeletal muscle [1] and mice lacking beta_2_‐adrenergic receptors exhibit reduced muscle mass and function [2]. Targeting the beta_2_‐adrenergic receptor with selective beta_2_‐agonists induces muscle hypertrophy, enhances muscle strength and improves glucose tolerance and insulin sensitivity after only a few weeks of treatment in humans [2, 3, 4, 5, 6, 7, 8]. Terbutaline, a highly selective and well‐tolerated beta_2_‐agonist clinically used in asthma, effectively increases muscle mass around 1–2 kg [5, 7] and improves insulin sensitivity by 25% following 4 weeks of treatment in otherwise healthy young individuals [9, 10]. Extending on these findings, we recently observed that terbutaline treatment shared some features of resistance training pertaining to the adaptive response of skeletal muscle [11]. However, the study was limited by a proteomic coverage of around 1100 proteins and utilized bulk muscle lysates, which do not consider the unique tissue organization of skeletal muscle.

Skeletal muscle tissue is highly heterogeneous, consisting of multiple cell types with a predominance of Type I and Type II muscle fibres, which are known to undergo distinct responses to physical activity, adrenergic stimuli and aging [4, 12, 13, 14, 15]. Recent advances in mass spectrometry (MS)–based proteomics now enable an in‐depth interrogation of the fibre type–specific adaptation from exceedingly small tissue amounts covering more than 2000 unique proteins [14, 16, 17, 18]. This allows the deciphering of the underlying distinct fibre‐type adaptations during beta_2_‐adrenergic stimulation and assessment of the overlap with protein‐wide remodelling induced by resistance training. Such knowledge is important to assess the potential of the beta_2_‐adrenergic receptor as a drug target for treating muscle atrophy.

In the present study, we harness an MS‐based proteomics workflow to interrogate the fibre type–specific responsiveness to beta_2_‐adrenergic stimulation and resistance training. We comprehensibly map the fibre type–specific protein‐wide adaptations induced by prolonged exposure to beta_2_‐adrenergic stimulation or resistance training and reveal that beta_2_‐adrenergic stimulation induces several resistance training‐like adaptations, particularly related to ribosomal biogenesis, despite imposing no mechanical load. Notably, beta_2_‐adrenergic stimulation increased ribosomal protein abundance uniformly across fibre types, whereas resistance training elicited a greater response in Type I fibres. In addition, S100A13 was upregulated across both interventions and fibre types, whereas MUSTN1 was regulated exclusively by resistance training. Functional experiments in C2C12 muscle cells demonstrated that both S100a13 and Mustn1 are essential for muscle growth.

## 2 Methods

### 2.1 Participants

Twenty‐five males gave their verbal and written informed consent and participated in the study. Data pertaining to body composition have been presented elsewhere [7]. The study complies with the guidelines of the 2013 Declaration of Helsinki and was approved by the Regional Ethics Committee of Copenhagen (H‐4‐2014‐002). In addition, the study was registered in Clinicaltrials.gov (trial identifier: NCT02557581). Participants were young (mean ± SD age: 24 ± 3), non‐obese (BMI: 23 ± 1 kg/m^2^), lean (body fat: 18% ± 4%), moderately trained (maximal oxygen uptake: 51 ± 5 mL O_2_/min/kg) and healthy males.

### 2.2 Training Intervention

Resistance training has been described in detail elsewhere [7]. Briefly, participants completed supervised training three times weekly for 4 weeks and had a compliance of 100% with the training protocol. Each training session was full body and consisted of two to four sets of leg press, lunges, leg extension, leg curls, bench press, incline bench press, low row, lateral pulldowns and military press. Load was continually adjusted between sets to reach volitional failure at ~10 repetitions, and sets were interspersed by 2 min of rest. After each training session, participants received a protein‐rich drink with carbohydrates (30 g of whey protein, Arla Foods, Viby J, Denmark; 35 g of carbohydrates, Maxim Sports Drink Orange, Orkla Care, Ishøj, Denmark) to stimulate postexercise protein synthesis.

### 2.3 Treatment With Beta_2_‐Adrenergic Agonist

Beta_2_‐adrenergic agonist treatment consisted of daily inhalation (8 × 0.5 mg, Bricanyl Turbuhaler, AstraZeneca, Cambridge, UK) which was supervised by daily video monitoring. Compliance with the treatment protocol was 100% [7].

### 2.4 Power Output During 30‐s Bike‐Ergometer Sprinting

Participants' peak and mean power output were assessed during a 30‐s sprint on a bike ergometer (Monark 839E; Monark Exercise AB, Vansbro, Sweden). Participants pedalled against a resistance of 6 N until a cadence of ~100 rpm was reached. Thereafter, ergometer resistance increased to 0.9 N/kg body mass, and participants pedalled as fast as possible for 30 s in a seated position. Power output during the 30‐s sprint was recorded using Monark software (Monark Anaerobic Test Software, v.3.3). Peak power output was determined as the highest power output at any recording time point, whereas mean power output was determined as the average power output during the entire sprint.

### 2.5 One‐Repetition Maximum (1RM) Testing

For participants in the resistance training group, 1RM leg extension was assessed after a 5‐min standardized warm‐up on a bike ergometer followed by 10–12 repetitions of leg extension with no resistance and then with a light load (~50% 1RM). Participants then performed sets of one repetition of increasing weight until the weight could not be lifted through the full range of motion. Three minutes of rest were provided between each successive attempt. For each exercise, the highest load successfully lifted was defined as 1RM. All 1RM determinations were made within five attempts.

### 2.6 Muscle Biopsy Collection

The skin above the belly of the m. vastus lateralis was anaesthetized with local anaesthesia (~2 mL Xylocain 2%; lidocaine without epinephrine, AstraZeneca, Denmark). A muscle biopsy (~150 mg w.w.) was sampled with a modified Bergström needle with suction through a ~2‐mm incision in the skin. Immediately after collection, the biopsy piece was rinsed in NaCl solution, frozen in liquid nitrogen and stored at −80°C until analysis.

### 2.7 Fibre Collection and Type Determination

Freeze‐dried muscle tissue biopsies were dissected with fine forceps under a microscope in a temperature and humidity‐controlled room (20°C and <20% relative humidity). Forty fibres were collected from each biopsy with an approximate length of ~1 mm. Fibres were placed in individual 0.5‐mL low‐bind Eppendorf tubes and stored at −20°C until further analysis. Upon completion of dissection, individual fibres were dissolved in 14 μL lysis buffer (1% sodium dodecyl sulphate [SDC], in 50‐mM Tris pH 8.5) and heated at 95°C for 10 min in a ThermoMixer during gentle shaking (650 rpm). Then, samples were centrifuged briefly in a benchtop centrifuge to ensure that all liquid collected at the bottom of the tube. Next, samples were sonicated (BioRuptor) in 30‐s on/off cycles for 15 min, followed by another centrifugation.

Fibre type was determined by dot blotting according to a modified protocol [19]. Briefly, polyvinylidene difluoride (PVDF; Millipore A/S, Copenhagen, Denmark) membranes were activated for 10 s in 96% ethanol and washed in transfer buffer >5 min. Activated PVDF membranes were collected, and excess transfer buffer was removed by gentle shaking of the membrane before placing it on a stack of five filter papers with the two top papers having been soaked in transfer buffer. PVDF membranes were allowed to dry for 1–2 min until no visible drops of transfer buffer remained. Then, 1 μL of fibre lysates were spotted in the same position for each individual lysate on two membranes with an eight‐channel multipipette, and membranes were removed from the filter paper and allowed to dry completely (~5 min) on a plastic lid. Membranes were then reactivated in 96% ethanol for 10 s, washed in transfer buffer for 5 min and blocked in 5% skim milk in Tris‐buffered saline (TBS) containing 0.1% Tween‐20 (TBST) for 15 min. Following blocking, membranes were washed three quick times in TBST and then incubated in primary antibody (MYH2: mouse igM, 1:200, A4.1519, DSHB, MYH7: mouse igM, 1:200, A4.840, DSHB; 1:200 dilution in TBST with 1% milk) at room temperature for 2 h during gentle movement. Subsequently, membranes were washed 2 × 5 min in TBST before incubation in horseradish peroxidase–conjugated secondary antibody (Goat Anti‐Mouse, Dako P0447, 1:20 000 dilution in TBST with 1% milk) at room temperature for 1 h during gentle moving. Membranes were then washed in 3 × 15 min in TBST and visualized with ECL (Millipore) and recorded with a digital camera (ChemiDoc MP Imaging System, Bio‐Rad Laboratories, USA).

### 2.8 Single‐Fibre Pool Sample Preparation

After dot blotting, fibres that stained positive for both MYH7 and MYH2, as well as fibres with no signal, were discarded. Pooled samples were collected using 10 μL from six fibres of each fibre type per participant and time point (i.e., preintervention and postintervention). The choice of six fibres was to ensure equal number of fibres per pooled sample, and the final number was determined by the lowest amount of Type II fibres across all participants, fibre types and time points. From each pooled sample, 50 μL was collected in a new lo‐bind Eppendorf tube and chloroacetamide (CAA) and dithiothreitol (DTT) were added to final concentrations of 40 and 10 mM, respectively, and incubated for 45 min at room temperature for reduction and alkylation. Then, samples were digested by addition of trypsin (Promega, Madison, Wisconsin) and LysC in enzyme:protein ratios of 1:100 and 1:500 for trypsin and LysC, respectively. Protein concentrations were estimated from a previous experiment with a similar sample preparation approach [20]. Digestion occurred overnight in a Thermomixer at 37°C and 800 rpm. Protein digestion was halted the following morning by the addition of 50 μL 2% trifluoroacetic acid (TFA) in isopropanol. Peptides were desalted using in‐house prepared single‐use reverse‐phase StageTips containing styrene divinylbenzene reverse‐phase sulfonate (SDB‐RPS) discs. Then, peptide concentration was measured with NanoDrop (Thermo Fisher Scientific) and volumes containing 200 ng of peptide were loaded in Evotips (Evosep) following manufacturer instructions prior LC–MS/MS analysis.

### 2.9 Library Preparation

An in‐house library with 5000 protein groups generated with 200 μg of protein from a pooled fibre lysate consisting of 1000 muscle fibres was used, prepared according to the procedure described above. Twenty micrograms of desalted peptides were fractionated using high pH reverse‐phase chromatography (HpH‐RP). Fractionation was carried out on a Kinetex 2.6‐μm EVO C18 100 Å, 150 × 0.3‐mm column manufactured by Phenomenex and using an EASYnLC 1200 System (Thermo) operating at 1.5 μL/min. Separation was accomplished using a 62‐min step gradient starting from 3% to 60% Solvent B (which consisted of 10‐mM TEAB in 80% acetonitrile) and Solvent A (containing 10‐mM TEAB in water). The total run time was 98 min, which included wash and column equilibration. Throughout the fractionation, peptides were eluted and collected every 60 s, obtaining 96 single fractions without concatenation. Finally, 200 ng of HpH‐RP fractionated peptides was loaded, concentrated and desalted on Evotips (Evosep) following the instructions provided by the manufacturer.

### 2.10 LC–MS/MS

Proteomics measurements were performed using LC–MS instrumentation consisting of an Evosep One HPLC system with a 21‐min gradient, coupled via electrospray ionization to a timsTOF SCP mass spectrometer (Bruker). Peptides were separated utilizing 8‐cm, 150‐μm ID columns packed with C18 beads (1.5 μm) (Evosep). Pooled single muscle fibre peptides were measured in DIA‐PASEF mode, while library fractions were measured using DDA‐PASEF. In brief, the DDA‐PASEF scan range encompassed 100–1700 *m/z* for both MS and MS/MS, and TIMS mobility range was set to 0.6–1.6 (V/cm^2^). Both TIMS ramp and accumulation times were configured to 100 ms, and 10 PASEF ramps were recorded for a total cycle time of 1.17 s. The MS/MS target intensity and intensity threshold were defined as 20.000 and 1.000, respectively. An exclusion list of 0.4 min for precursors within 0.015 *m/z* and 0.015‐V/cm^2^ width was also activated. For DIA‐PASEF, the scan range was established at 400–1000 (*m/z*), the TIMS mobility range to 0.64–1.37 (V/cm^2^) and ramp and accumulation times were both set to 100 ms. A short‐gradient method was used, which included eight DIA‐PASEF scans with three 25‐Da windows per ramp, resulting in an estimated cycle time of 0.95 s.

### 2.11 MS Data Processing

Library files were processed using the MS‐Fragger tab within Fragpipe v.19.0 under the SpecLib workflow with default settings, including a minimum peptide length of seven amino acids and a maximum of two missed cleavages allowed [21]. Spectra were searched against a human reviewed FASTA from Uniprot (March 2022, 20 410 entries), and the output library contained a total of 5350 protein groups and 84 383 precursors. Experimental raw MS files were analysed using DIA‐NN version 1.876 [22] in a library‐based manner against the MS library just described. Quantification of protein groups was based on proteotypic peptides, neural network was set to double‐pass mode, the quantification strategy was set to ‘Robust LC (high accuracy)’ and the match between runs options was enabled. The rest of the parameters remained as default, which included precursor FDR set to 1% and peptide length of 7–30 amino acids. The resulting dataset contained 2320 protein groups (henceforth ‘proteins’) before filtering. The protein groups file from the DIA‐NN output was annotated and utilized for downstream bioinformatics analysis.

### 2.12 Bioinformatics

Downstream bioinformatic analysis was conducted in the R environment (v.4.5.1, Foundation for Statistical Computing, Vienna, Austria). Twelve samples (out of 92) were excluded from data analysis based on MYH2 or MYH7 not contributing the majority myosin isoform in their respective sample pools. This was to ensure that contamination of samples by impure fibres did not confound the analysis. In cases where only the pre sample was contaminated for a given participant, the post sample within the same fibre type was also excluded. The final dataset thus consisted of eight participants in the resistance training group and 13 participants in the beta_2_‐agonist group. Data were filtered for 70% valid values in at least one fibre type, resulting in 2109 proteins. Values were log_2_‐transformed and median scaled, followed by annotation with gene ontology terms (GO:BP, GO:CC and GO:MF) and keywords from the UniProt database. Mitochondrial proteins were annotated with MitoCarta 3.0.

### 2.13 Differential Expression Analysis

Statistical analysis of proteomics data was conducted with the limma package (v.3.62.2). A linear model was fit using protein‐wise log_2_‐transformed intensities, incorporating both time (pre/post) and intervention group (beta_2_/resistance) or fibre type (Type I/Type II) as fixed effects. Contrasts were specified to evaluate the main effects of time for each fibre type for each intervention group, within‐group effects and interactions (intervention group × time). Individual participant ID was included as a blocking factor to account for within‐subject correlation using limma::duplicateCorrelation. Models were fit using limma::lmFit, and statistical inference was performed using empirical Bayes moderation (limma::eBayes). Because of the exploratory nature of identifying new biomarkers of importance for hypertrophy, we adjusted *p* values using a *π* score, which was calculated based on statistical significance (*p* value) and biological relevance (log_2_fold change) according to *π* = *p* value^|log2foldchange|^ [23]. *p* values adjusted for multiple comparisons, as described by Benjamini–Hochberg and Storey and Tibshirani [24], are supplied in Table S3.

### 2.14 Principal Component Analysis

Principal component analysis was performed with the R package pcaMethods (v.1.98). For PCA purposes, data were scaled and centered, and filtered for proteins with no missing values (total of 1462 proteins).

### 2.15 Gene Set Enrichment Analysis

Gene set enrichment analysis was conducted with the R package clusterProfiler (v.4.14.6). All quantified proteins (total number: 2109) were ranked according to log_2_fold change, and enrichment was assessed against gene ontology categories (GO: Biological Process and Cellular Component) using the clusterProfiler::gsea() function. Gene sets were tested for enrichment at the extremes of the ranked list without applying a hard significance threshold. *p* values were adjusted for multiple testing using the Benjamini–Hochberg procedure.

### 2.16 Cell Culture and Differentiation of C2C12 Cells

C2C12 muscle cells were obtained from American Type Culture Collection (ATCC CRL‐1772) and grown in Dulbecco's modified Eagle's medium (DMEM), supplemented with 100 U/mL penicillin, 100 μg/mL of streptomycin and 10% fetal bovine serum. Subculturing was done by trypsinization, and cells were cultured with a density of 0.1 × 105 on PhenoPlate 96‐well plates for immunofluorescence or 0.1 × 106 on 12‐well plates for gene expression analysis. When cells reached ~80% confluence, differentiation was induced by switching to DMEM, supplemented with 100 U/mL penicillin, 100 μg/mL of streptomycin and 2% fetal bovine serum. The medium was changed every other day, and the cells were incubated at 37°C in a humidified 5% CO_2_ environment for 7 days.

### 2.17 Cell Transfection

Beginning at the second day of differentiation, cells were transfected with either siRNA Silencer Select S100a13 (4390771, Thermo Fisher Scientific, Waltham, MA, USA), ON‐TARGETplus Mouse Mustn1 siRNA SMART pool (L‐058444‐01‐0005, Dharmacon) or siRNA Silencer Select Negative Control (4390843, Invitrogen) using Lipofectamine RNAiMAX (13778‐030, Invitrogen). Both Lipofectamine RNAiMAX and siRNAs were diluted in Opti‐MEM Medium (31985070, Gibco) according to manufacturer instructions. Diluted siRNA and Lipofectamine RNAiMAX were mixed (1:1 ratio) and incubated at room temperature according to manufacturer instructions. siRNA‐lipid complex was then added to cells. Forty‐eight hours following transfection, cell medium was replaced with complete differentiation medium.

### 2.18 RNA Extraction, Reverse Transcription and qPCR

Forty‐eight hours after transfection, cells were lysed with QIAzol lysis reagent (QIAGEN, Germany), and the total RNA was extracted using the miRNeasy Mini Kit (QIAGEN) according to the manufacturer's procedure. RNA concentration was measured with a Qubit 3 (Thermo Fisher Scientific Waltham MA USA). For reverse transcription, 1 μg of RNA was retrotranscribed using the High‐Capacity cDNA Reverse Transcription Kit (Thermo Fisher Scientific, Waltham, MA, USA). qPCR analysis was performed using SYBR green (PowerUp SYBR Green Master mix, Thermo Fisher Scientific, Waltham, MA, USA). S100a13 and Mustn1 gene expression values were normalized to the expression level of 18S. The fold changes were obtained by the ^ΔΔ^Ct method, using cells treated with siRNA negative control as a control condition. The sequences of primers used in the study are listed in Table 1.

**TABLE 1:** List of primer sequences.

Gene	Sequence (5′‐3′)
*18s*—Forward	CATGGCCGTTCTTAGTTGGT
*18s*—Reverse	CGCTGAGCCAGTCAGTGTAG
*S100a13*—Forward	GCTGTGTTGGGATGGCTAGT
*S100a13*—Reverse	AAGCAGGAGCTGATGTCCAC
*Mustn1*—Forward	AAGAAGAAGCGGCCCCCT
*Mustn1*—Reverse	CTTTGGGCTTCTCAAAGAC

### 2.19 Immunofluorescence and Image Analysis

Ninety‐six hours after transfection, myotubes were washed with 1× PBS and fixed in 4% paraformaldehyde for 10 min at room temperature. Then, cells were permeabilized using 0.5% Triton‐X 100 for 30 min and blocked for 30 min in PBS with 5% BSA at room temperature. Afterward, myotubes were incubated in primary antibody overnight at 4°C. The following day, cells were washed three times with 1× PBS and incubated with fluorescence‐labelled secondary antibody. The primary antibody used was skeletal muscle myosin (MYH1; cat. M4276, Sigma‐Aldrich). The secondary antibody used was a goat antirabbit conjugated with Alexa Fluor 546 (cat. A11030, Invitrogen). Nuclei were counterstained using DAPI.

Images were acquired using the automatic confocal fluorescence microscopy Operetta CLS (Revvity), with a 20× water objective. Briefly, nuclei and myotubes were automatically segmented considering the DAPI and Alex Fluor 546 (myosin) positive channels, respectively. Then, the nuclei inside the myotubes were automatically counted. To obtain the fusion index, the nuclei in the myotubes were divided by the total number of nuclei and multiplied by a hundred. Image analysis was performed using Harmony Software (Revvity).

### 2.20 Statistics

For univariate correlation analyses, the Pearson product–moment correlation coefficient was employed in SPSS (v.29, IBM Software, Chicago, Illinois, USA). For summed abundances of proteins belonging to specific biological processes or pathways, a linear mixed model was performed using the R package lme4 with participant as a random factor and time (pre/post) and intervention (beta_2_/resistance) as fixed factors. Data are presented with *p* values to represent probability.

## 3 Results

### 3.1 Deep Proteome Analysis of Slow and Fast Muscle Fibres

**FIGURE 1:**
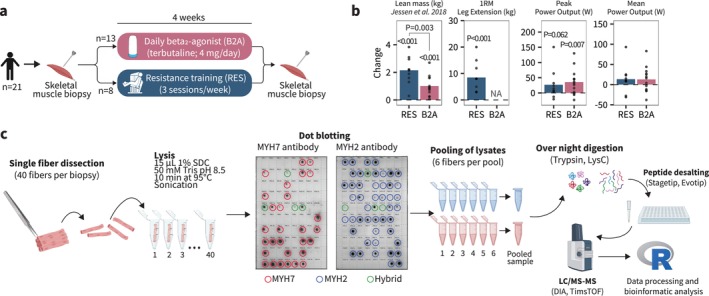
Fibre type–resolved proteomics enables deep profiling of beta_2_‐adrenergic and resistance training‐induced adaptations. (a) Study design in which pooled fibres from 21 participants were analysed. Participants completed either 4 weeks of resistance training or inhalation of a beta_2_‐adrenergic agonist. (b) Changes in functional outcomes and lean mass. (c) Preparation of fibre‐type pools. Fibres were dissected from freeze‐dried muscle tissue and lysed in SDC buffer before dot blotting for fibre‐type determination with MYH2 and MYH7 antibodies. Fibres coexpressing myosin isoforms were discarded. Then, six lysates from each fibre type from each participant were pooled into a pooled sample. Pooled samples were digested with trypsin and LysC overnight, followed by SDB‐RPS StageTips and loaded into Evotips before analysis by DIA‐PASEF LC/MS–MS in a TimsTOF. Protein identification in DIA‐NN and bioinformatics analysis in R. B2A: beta_2_‐adrenergic stimulation; RES: resistance training. Lean mass in panel (b) has been presented elsewhere (beta_2_‐adrenergic agonist group [habitually active] and placebo group [resistance training]) in [7]. 1RM: 1 repetition maximum.

**FIGURE 2:**
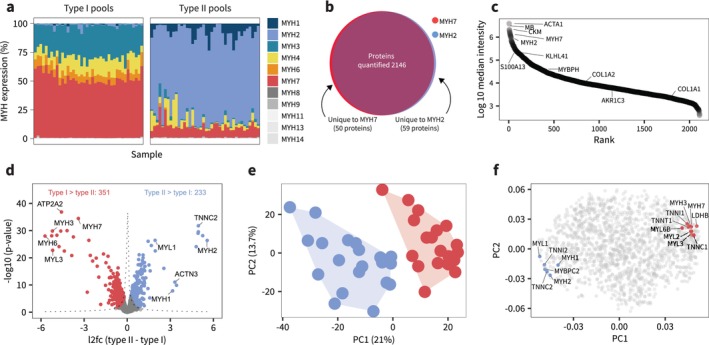
Distinct proteomic landscapes define Type I and Type II muscle fibres. (a) MYH isoform distribution in pooled samples. (b) Venn diagram of identified proteins and number of proteins unique to fibre types after filtering for 70% valid values in at least one time point for each fibre type. (c) Ranked raw intensities of all quantified proteins. (d) Volcano plot showing differences in individual protein abundances between Type I and Type II fibres. (e) Principal component analysis (PCA) on log_2_‐transformed scaled and centered data showing separation of baseline samples. For the purposes of PCA, data were filtered for 100% valid values (1462 proteins) before analysis with R package pcaMethods. (f) Main drivers of separation in PCA (principal component loadings). These were primarily related to isoforms of myosin, myosin light chain and troponin.

We utilized freeze‐dried vastus lateralis muscle tissue from 21 moderately trained male participants collected before and after a 4‐week intervention consisting of either whole‐body resistance training (*n* = 8) or daily inhalation of beta_2_‐adrenergic agonist, terbutaline (4 mg/day, *n* = 13; Figure 1a) [7]. Leg lean mass [25] and whole‐body lean mass [7] (Figure 1b), as assessed by dual‐energy X‐ray absorptiometry, increased with both beta_2_‐adrenergic stimulation and resistance training [7], although to a greater extent with resistance training (time × treatment interaction effect: *p* = 0.003). Leg extension 1RM increased by 12.5% with resistance training (*p* < 0.001). Peak‐power output during a 30‐s maximal bicycle ergometer sprint increased in both treatments with no between‐group differences (time × treatment interaction effect: *p* = 0.644), whereas mean power output did not change with either treatment (*p* = 0.185 and *p* = 0.146 for resistance training and beta_2_‐adrenergic stimulation, respectively; Figure 2b).

From each biopsy, we dissected 40 single fibres under a stereomicroscope and determined fibre type by antibody‐based dot blotting for expression of MYH7 (Type I) and MYH2 (Type IIa), respectively. We created pooled samples of six fibres per time point and fibre type for each participant (Figure 1c). Based on recent evidence challenging the dogma that Type IIx fibres are phenotypically distinct from Type IIa fibres [20], as well as the overall low abundance of MYH1 (primary myosin isoform of Type IIx fibres; Figure 2a), we made no distinction between Type IIa and IIx fibres in our analysis. We also excluded fibres coexpressing MYH7 and MYH2 isoforms to attain pure fibre‐type pools for genuine assessment of fibre type–specific adaptations (Figure 2a). Using a short chromatographic gradient (21 min) coupled with a highly sensitive timsTOF mass spectrometer and DIA‐PASEF data acquisition method [26], we quantified 2307 protein groups (henceforth ‘proteins’, Table S1). After filtering for 70% valid values, 50 proteins were unique to Type I fibres, and 59 proteins were unique to Type II fibres (Figure 2b), with a total proteomic range spanning ~5 orders of magnitude (Figure 2c). Between fibre types, 351 proteins had a significantly higher abundance in Type I fibres, whereas 233 were higher in Type II fibres (Figure 2d). Fibre types separated clearly along the first component in principal component analysis (Figure 2e), which accounted for 21% of the total variance. This separation was driven by the most differentially abundant proteins, mainly comprising myosin isoforms, the actin cytoskeleton, myosin light chains and troponins (Figure 2f).

### 3.2 Resistance Training and Beta_2_‐Adrenergic Stimulation Induce Highly Fibre Type–Dependent Responses

**FIGURE 3:**
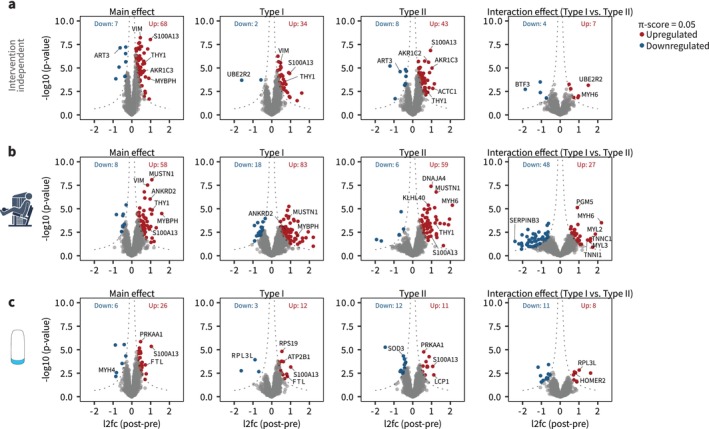
Resistance training and beta_2_‐adrenergic stimulation induce proteomic landscape changes in a highly fibre type–dependent manner. (a–c) Volcano plots of log_2_fold changes independent of intervention group (a), with resistance training group (b) and beta_2_‐adrenergic stimulation (c). Plots are of main effect (independent of fibre type), for fibre types individually and for interaction effect (fibre type × time). A significance score (*π*; combining log_2_fold change and *p* value) of 0.05 was used.

Having established pure fibre‐type pools, we proceeded to investigate the overall effect of the intervention in the proteomic landscape with differential abundance analysis. Independent of the intervention group or fibre types, 75 proteins were regulated by the intervention (Figure 3a; main effect), whereas only 11 proteins were regulated in a fibre type–specific manner (Figure 3a; interaction effect). Both resistance training and beta_2_‐adrenergic stimulation modified overall abundances of numerous proteins (Figure 3b,c; main effect, Table S3). While the regulation was generally greater with resistance training, the number of regulated proteins was approximately equal between fibre types within each intervention group. Thus, resistance training regulated 101 proteins in Type I fibres and 65 proteins in Type II fibres, while the corresponding number was lower with beta_2_‐adrenergic stimulation (15 and 23 in Type I and Type II fibres, respectively). In both interventions, several proteins exhibited fibre type–specific regulation, with resistance training in particular inducing greater increases in the protein abundance in Type II fibres, which included primarily sarcomeric proteins (Figure 3b; interaction effect).

**FIGURE 4:**
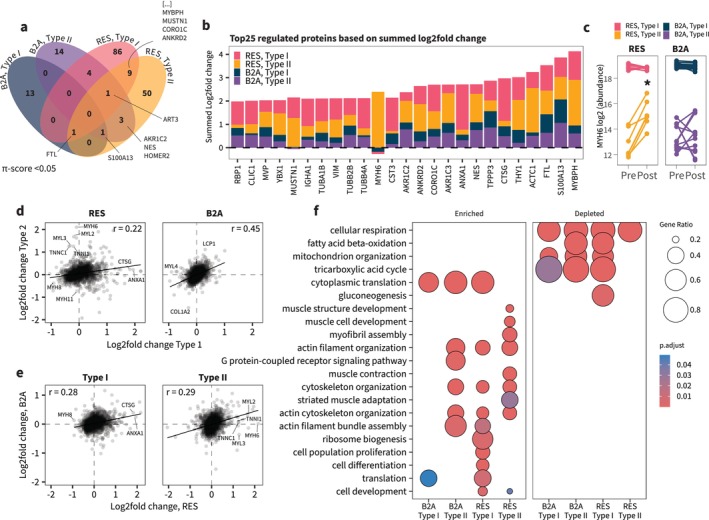
Fibre type–specific proteome remodelling reveals shared and divergent responses. (a) Venn diagram showing overlap in significantly regulated proteins. (b) Bar chart showing the top 25 summed median log_2_fold changes. (c) Individual changes in MYH6 protein abundances. (d) Pearson product moment correlation of log_2_fold changes in Type I versus Type II fibres with each intervention. (e) Pearson product moment correlation of log_2_fold changes with resistance training (RES) and beta_2_‐adrenergic stimulation (B2A) within each fibre type. (f) Gene set enrichment analysis of gene ontology biological processes (GO:BP). B2A: beta_2_‐adrenergic stimulation; RES: resistance training. Complete list of enriched and depleted terms is available in Table S4.

Regulated proteins revealed a remarkable fibre type–dependent response at the protein level for both resistance training and beta_2_‐adrenergic stimulation (Figure 4a). Only 7% (11 out of 155) of the proteins significantly regulated by resistance training were shared between fibre types, compared to 3% (1 out of 37) for beta_2_‐adrenergic stimulation. The shared protein for beta_2_‐adrenergic stimulation was S100A13, a Ca^2+^‐binding protein involved in the secretion of IL‐1α and FGF1 and known to be highly regulated by exercise training [11, 14, 27]. No proteins were regulated across all conditions (i.e., both fibre types for both beta_2_‐adrenergic stimulation and resistance training interventions).

We further assessed log_2_fold changes of the 25 proteins with the highest overall changes in protein abundance (i.e., combining both interventions and fibre types; Figure 4b). While resistance training generally induced greater abundance changes, S100A13 consistently increased to the same extent in both fibre types and interventions, whereas other proteins were exclusively regulated by resistance training. For example, resistance training increased MYBPH and MUSTN1 abundance in both fibre types, whereas expression of MYH6 only increased in Type II fibres and not with beta_2_‐adrenergic stimulation (Figure 4c). MYH6 is typically associated with Type I fibres (and indeed is much more abundant in Type I fibres in the present study, Figure 4c) and may suggest a transition of Type II fibres towards a myosin phenotype more similar to Type I fibres with resistance training. Given their distinct regulatory patterns and potential roles in muscle remodelling, we selected S100A13 and MUSTN1 for further functional validation in cell‐based assays (see Figure 7).

To assess the overall fibre‐type dependence on adaptations to resistance training and beta_2_‐adrenergic stimulation, we correlated the log_2_fold changes in Type I and Type IIa fibres for all proteins within each intervention (Figure 4d,e). Although changes were on average greater with resistance training, they were notably less correlated between fibre types (*r* = 0.21) compared with the changes observed with beta_2_‐adrenergic stimulation (*r* = 0.41). With resistance training, proteins related to muscle contraction responded differently in Type I and Type II fibres. For example, MYH6, myosin light chains MYL2 and MYL3 and troponins TNNC1 and TNNI1 appeared to exhibit more pronounced upregulation in Type II fibres, but not Type I, in agreement with the results from the fibre type × time interaction analysis. Thus, considering global protein changes, the observed proteome remodelling is more influenced by muscle fibre type upon resistance training than under beta_2_‐adrenergic stimulation.

We performed gene set enrichment analysis within each fibre type and intervention and discovered a clear enrichment of cytoplasmic translation processes. Unexpectedly, while this was evident for only Type I fibres with resistance training (Figure 4f), both fibre types were enriched for this term in response to beta_2_‐adrenergic stimulation (Figure 4f). Also of particular importance was the depletion of terms related to mitochondrial protein expression in all fibre types in response to both beta_2_‐adrenergic stimulation and resistance training. While this adaptation has been reported for both resistance training and beta_2_‐adrenergic stimulation [6, 28] in whole muscle lysate, here, we describe for the first time the fibre type–specific adaptations and comparison between two hypertrophy‐inducing interventions.

### 3.3 Resistance Training and Beta_2_‐Adrenergic Stimulation Increase Ribosomal but Decrease Mitochondrial Proteins in a Fibre Type–Specific Manner

**FIGURE 5:**
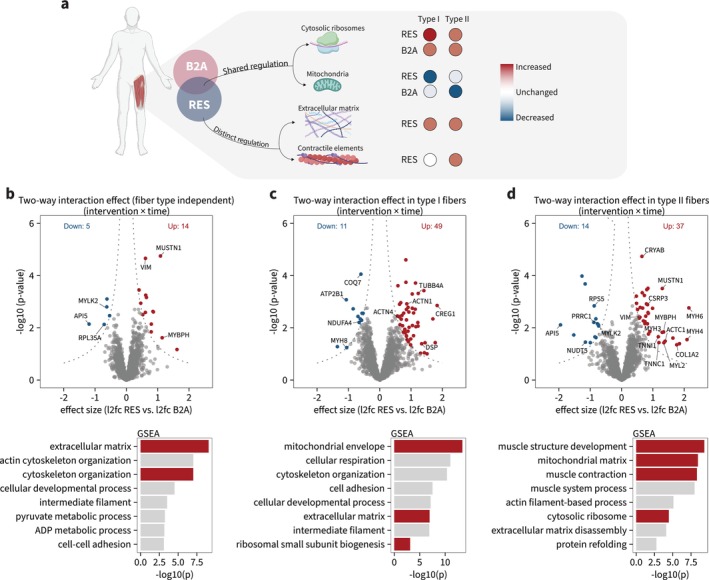
Resistance training induces distinct structural adaptations not mimicked by beta_2_‐adrenergic stimulation. (a) Schematic presentation of main findings. (b) Volcano plot showing intervention × time interaction effects for both fibre types combined and selected terms from gene set enrichment analysis. (c) Volcano plot showing intervention × time interaction effects for Type I fibres and selected terms from gene set enrichment analysis. (d) Volcano plot showing intervention × time interaction effects for Type II fibres and selected terms from gene set enrichment analysis.

Having identified biological processes regulated within each intervention, we proceeded to compare the proteomic regulation between interventions (Figure 5a). In a fibre type–independent analysis, resistance training increased 14 proteins compared with beta_2_‐adrenergic stimulation, and gene set enrichment analysis revealed intervention differences in extracellular matrix composition and cytoskeletal organization (Figure 5b). Upon stratifying by fibre type, our analysis revealed differences between interventions in mitochondrial and ribosomal proteins across both fibre types (Figure 5c,d), as well as in sarcomeric proteins specifically in Type II fibres (Figure 5d).

**FIGURE 6:**
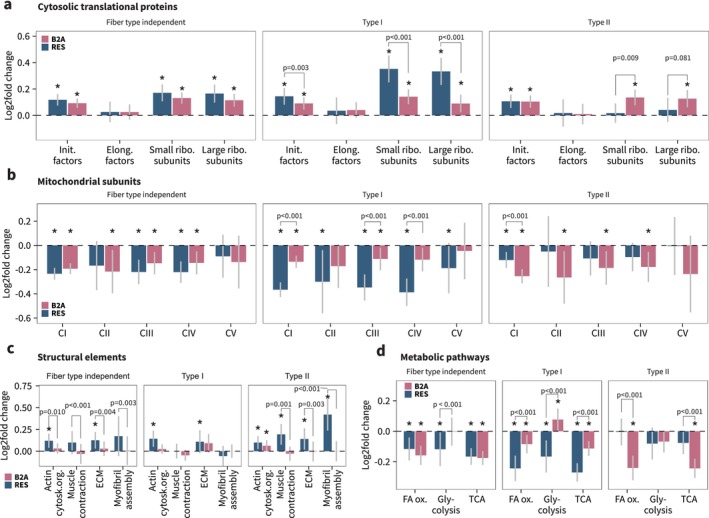
Resistance training and beta_2_‐adrenergic stimulation improve translational capacity but impair mitochondrial content in a fibre type–specific manner. (a) Mean log_2_fold changes of proteins annotated as subunits of small and large ribosomal subunits (annotated with GO:CC, small: GO:0022627, large: GO:0022625), as well as initiation and elongation factors (annotated with GO:BP, initiation factors: GO:0006413, elongation factors: GO:0006414). (b) Mean log_2_fold changes of proteins annotated as subunits of oxidative phosphorylation Complexes I–V. Annotation with MitoCarta 3.0. (c) Mean log_2_fold changes of proteins involved in actin cytoskeletal organization (GO:0030036), muscle contraction (GO:0006936), extracellular matrix (ECM; GO:0031012) and myofibril assembly (GO:0030239). (d) Mean log_2_fold changes of proteins involved in fatty acid beta oxidation (FA ox.; GO:0006635), glycolysis (GO:0061621) and tricarboxylic acid cycle (TCA; GO:0006099).

To further compare shared features of beta_2_‐adrenergic stimulation and resistance training in these specific processes, we examined summed protein abundances of selected protein categories. While proteins related to translational machinery were overall increased with beta_2_‐adrenergic stimulation, the effect was lower than with resistance training (Figure 6a). However, whereas resistance training led to a greater increase in translation initiation factors and large ribosomal subunits in Type I fibres, beta_2_‐adrenergic stimulation caused equal increases across both fibre types, with changes in Type II fibres larger than those seen with resistance training for small ribosomal subunit proteins (Figure 6a).

To determine changes in mitochondrial protein content, we assessed proteins belonging to complexes of the electron‐transport chain. Both beta_2_‐adrenergic stimulation and resistance training decreased the overall content of all complexes to largely the same extent (fibre type–independent analysis, Figure 6b). Consistent with changes in translational proteins, the response was comparable between the two interventions in Type II fibres, though beta_2_‐adrenergic stimulation caused greater decreases in complex CI. In contrast, the decrease in mitochondrial complexes was greater with resistance training in Type I fibres (Figure 6b).

In line with between‐intervention gene set enrichment analysis, components of the actin cytoskeleton (largely comprised of alpha‐actinins) and extracellular matrix (such as FBN1, FN1, FBLN5 and several collagens) were not increased by beta_2_‐adrenergic stimulation, whereas they were increased in both fibre types with resistance training (and in particular Type II fibres, Figure 6c). Moreover, beta_2_‐adrenergic stimulation did not increase proteins related to muscle contraction (e.g., myosin and titin isoforms) and myofibril assembly (such as FLII, MYH6, LMOD2 and LMOD3), while these were highly regulated with resistance training in Type II fibres only (Figure 6c).

Several metabolic processes were also affected by both interventions. Beta_2_‐adrenergic stimulation led to marked reductions in proteins related to fatty acid oxidation and the tricarboxylic acid (TCA) cycle, particularly in Type II fibres, while increasing proteins involved in glycolysis in Type I fibres (Figure 6d). In contrast, resistance training caused no changes in these processes in Type II fibres but led to significant decreases in fatty acid oxidation, TCA cycle and glycolysis proteins in Type I fibres (Figure 6d).

### 3.4 S100A13 and MUSTN1 Are Potential Mediators of Muscle Hypertrophy

**FIGURE 7:**
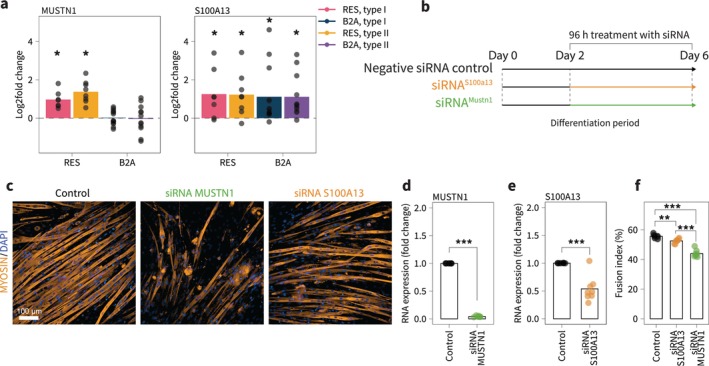
Mustn1 and S100a13 are important for cell growth. (a) Log_2_fold changes for MUSTN1 and S100A13 in human muscle samples. (b) Overview of cell experiment. C2C12 cells were treated with either small interfering RNA (siRNA) for Mustn1 or S100a13 at Day 2 of differentiation. (c) Representative pictures of myofibers at Day 6 of differentiation. (d) mRNA expression of Mustn1. (e) mRNA expression of S100a13. (f) Fusion index (number of nuclei in myotubes divided by total number of nuclei). Stars denote *p* value for comparisons shown by brackets (*p < 0.05, **p < 0.01, ***p < 0.001).

Having identified several proteins of potential relevance for muscle growth with resistance training and beta_2_‐adrenergic stimulation, we selected S100A13 and MUSTN1 for functional validation based on their distinct regulatory patterns and biological relevance regardless of *p*‐value adjustment approach (Table S3). S100A13 was the only protein consistently upregulated across both interventions and fibre types, suggesting a potential shared role in hypertrophic signalling. Although implicated in many biological processes, such as export of FGF1 [29] and IL1‐α [30] and tumour angiogenesis [31], the exact function of S100A13 in skeletal muscle remains elusive. In contrast, MUSTN1 was exclusively upregulated by resistance training, pointing to a training‐specific mechanism (Figure 7a). MUSTN1, expressed mainly in myofibers and satellite cells [32], is an essential regulator of muscle development based on evidence from several different species. For example, in vivo knock‐down of MUSTN1 impairs muscle force development in mice [33], and the protein is a target of interest to increased meat production in livestock [34, 35, 36].

To further explore their functional relevance, we performed knock‐down experiments in C2C12 cells starting at the second day of differentiation (Figure 7b) and used confocal imaging to visualize myotube assembly (Figure 7c). siRNA treatment resulted in a 96% reduction in Mustn1 mRNA expression (*p* < 0.001; Figure 4d) and a 52% reduction in S100a13 mRNA expression (*p* < 0.001; Figure 7e). Both knock‐downs impaired myotube development, with particularly pronounced effects of Mustn1 silencing. Fusion index, representing the proportion of nuclei incorporated into myotubes relative to the total number of nuclei, decreased by 21% (*p* < 0.001) and 5% (*p* = 0.002) with knock‐down of Mustn1 and S100a13, respectively (Figure 7f).

## 4 Discussion

Developing new therapies to counteract muscle atrophy and enhance muscle mass is essential, especially for individuals unable to engage in resistance training. In this study, we used a state‐of‐the‐art MS workflow to map fibre type–specific adaptations in response to beta_2_‐adrenergic stimulation and resistance training. We show that fibre type is a key modulator in the differential effects of resistance training and beta_2_‐adrenergic stimulation, highlighting the importance of considering fibre type–specific analysis to deeply understand the intricacies of exercise and drug‐induced protein remodelling in a heterogeneous tissue such as skeletal muscle.

The key shared adaptation between beta_2_‐adrenergic stimulation and resistance training pertained to an increase in ribosomal small and large subunits and initiation factors. This finding is of importance because ribosomal protein content plays a pivotal role in supporting skeletal muscle hypertrophy [37]. While it is not clear whether the increase in ribosomal biogenesis is a mechanism or a consequence of beta_2_‐adrenoceptor mediated hypertrophy, it is remarkable that beta_2_‐adrenergic stimulation induced greater increases in ribosomal subunit proteins than resistance training in Type II fibres. For example, RPL35 increased ~1.4‐fold in Type II fibres with beta_2_‐adrenergic stimulation and ~1.2‐fold with resistance training. While the magnitude of individual ribosomal subunit changes may appear modest, the overall consistency across fibre types within just 4 weeks is a compelling finding for the use of beta_2_‐agonists for inducing muscle hypertrophy and improving muscle function.

Some pharmacological strategies to induce muscle hypertrophy fail to induce functional improvements [38]. Our beta_2_‐agonist–treated cohort improved peak power during bike‐ergometer sprinting to the same extent as resistance training, suggesting that even some of the functional adaptations of the early stages of resistance training are mimicked, as also shown previously [5, 39]. Interestingly, this effect occurred despite no apparent adaptations in contractile proteins in the beta_2_‐agonist–treated group, contrasting that of resistance training. On the other hand, the beta_2_‐agonist–treated group exhibited adaptations in several proteins uniquely involved in excitation–contraction coupling and sarcolemma‐cytoskeleton coupling. For example, ATP2B1, which has been linked to muscle relaxation kinetics by regulating cytosolic calcium gradients [40], increased in the beta_2_‐agonist–treated group, as did the structural protein EZR, which is involved in sarcolemma‐cytoskeleton coupling [41]. The combined hypertrophic and functional effects of beta_2_‐agonist are particularly relevant for populations where capacity for physical training may be limited, such as in patients suffering from sarcopenia, cachexia and other clinical populations where loss of muscle mass, function and consequently quality of life, is a hallmark feature [42]. Beta_2_‐agonists may also benefit those capable of low‐level activity by amplifying the adaptive response to exercise, as studies combining beta_2_‐adrenergic stimulation with resistance training demonstrate additive muscle hypertrophy [7, 8].

Mitochondrial protein abundances were markedly decreased following beta_2_‐adrenergic stimulation, which could raise concerns regarding the preservation of metabolic muscle quality. However, similar reductions were also observed with resistance training, which is known to enhance metabolic health, suggesting that these changes do not reflect negative metabolic effects. For example, mitochondrial mass‐specific respiratory function (i.e., adjusted for total mitochondrial protein abundance) is not impaired by prolonged beta_2_‐adrenergic stimulation [6] or resistance training [43]. Instead, the observed decrease likely reflects a relative dilution of mitochondrial proteins due to the preferential synthesis of non‐mitochondrial proteins, rather than a true decline in mitochondrial content.

While beta_2_‐adrenergic stimulation mimicked adaptations to the translational machinery, we found that some adaptations were unique to resistance training. This was largely reflected in increased abundances of extracellular matrix proteins and contractile elements. Specifically, increases in myosin isoforms (particularly MYH6), myosin light chains, titin and extracellular matrix proteins were distinct adaptations of resistance training. Given the role of the extracellular matrix in lateral force transmission [44] and myosins for force development, the mechanical load from resistance training is likely necessary for driving certain structural adaptations and demonstrates a divergent adaptation that may not be achievable by targeting the beta_2_‐adrenergic receptor alone. In other words, additional measures are likely required to achieve the full functional benefits offered by mechanical load–induced hypertrophy.

Skeletal muscle fibre types respond to exercise training in a highly heterogeneous manner. With endurance training, it was recently reported that less than 10% of the 377 regulated proteins were shared between fibre types [16]. Here, we extend these findings and report that resistance training induced a ~7% overlap in regulated proteins between Type I and Type II fibres and in addition show that beta_2_‐adrenergic stimulation displayed an equally low ~3% overlap. Moreover, both interventions induced several proteins that responded differently between fibre types. Particularly for resistance training, fibre‐type differences were striking for sarcomeric and contractile proteins increasing almost exclusively in Type II fibres. These findings add to the growing body of evidence demonstrating fibre‐type heterogeneity, not just between fibres but also in response to cellular stress. This is of particular importance for mapping adaptations to treatments aimed at preserving muscle function in patients with atrophic conditions. For example, beta_2_‐adrenergic stimulation in the present study induced consistent effects on ribosomal abundances across fibre types, whereas resistance training mainly affected ribosomal and mitochondrial protein abundances in Type I fibres. Given that human muscle composition typically follows a roughly 1:1 distribution of Type I and Type II fibres, it is critical to target both fibre types to optimize treatment effectiveness.

A novel finding of the present study was the upregulation of S100A13. While exercise training has previously been shown to increase S100A13 abundance [27], we show that this is not confined to endurance training, but also resistance training, and across fibre types. Notably, this substantial increase was also seen with beta_2_‐adrenergic stimulation, indicating that S100A13 is upregulated independently of mechanical load. In our follow‐up experiments, we further confirmed S100A13's importance in regulation of muscle growth, as S100a13 knock‐down decreased fusion index and attenuated myotube formation in C2C12 muscle cells. In contrast, we found that MUSTN1 protein abundance was strongly upregulated by resistance training, but not with beta_2_‐adrenergic stimulation. While our follow‐up studies confirmed that MUSTN1 likely plays a role in muscle growth, our data suggest that MUSTN1 may be stimulated by mechanical load but cannot be stimulated via the beta_2_‐adrenergic receptor. Notably, while a previous study in rodents found that acute exercise preferentially increases Mustn1 in Type I fibres [45], our findings demonstrate that in human skeletal muscle resistance training induces robust elevations of MUSTN1 protein expression across fibre types, suggesting conserved yet context‐dependent regulation of this growth‐associated protein. Collectively, our findings suggest that S100A13 and MUSTN1 are important for regulating muscle mass in response to stress (S100A13 mechanical and beta_2_‐adrenergic; MUSTN1: only mechanical) and that only S100A13 is mimicked by beta_2_‐adrenergic stimulation.

While our findings offer new insights pertaining to the beta_2_‐adrenergic as a target for muscle remodelling, several limitations should be acknowledged. First, the intervention duration of 4 weeks may not have captured the full extent of structural remodelling, particularly of extracellular matrix and contractile elements, and studies of longer duration are required to establish the efficacy of longer treatment durations. Second, as we discarded fibres staining positive for both MYH2 and MYH7, we are unable to make inferences about protein‐wide adaptations in hybrid fibres. Third, our study was limited to a relatively small sample of young, healthy males and warrants extension to older populations, including patients suffering from sarcopenia and cachexia, to provide even stronger translational evidence for the use of beta_2_‐adrenergic stimulation as a therapeutic option in muscle wasting conditions. Lastly, given that resistance training may induce sex and fibre type–specific proteome remodelling [14], a mixed sex population would have increased generalizability of our findings. Nevertheless, the response in muscle hypertrophy to beta_2_‐adrenergic stimulation does not seem to differ between sexes based on previous studies [6, 46].

In conclusion, our study uncovers fibre type–specific insights into how beta_2_‐adrenergic stimulation may induce several key protein‐wide adaptations of resistance training in the absence of mechanical load. In addition, we highlight S100A13 as part of a common regulatory network for muscle growth induced by both beta_2_‐adrenergic stimulation and resistance training. While some features related to muscle cell structure are unique to resistance training, the overlap in ribosomal adaptation in addition to the hypertrophic and functional effects incurred by beta_2_‐adrenergic stimulation suggests that the beta_2_‐adrenergic receptor may be a potential target to counter muscle atrophic conditions. This offers a potential pharmacological approach for patients unable to engage in resistance training and encourages further exploration into clinically relevant patient groups.

## Conflicts of Interest

The authors declare no conflicts of interest.

## Supporting information


**Data S1:** Supporting Information.

**Figure S1:** Data integrity and fibre‐type purity.

**Table S1:** Protein identifications.

**Table S2:** Metadata.

**Table S3:** Limma results.

**Table S4:** Gene set enrichment analyses.

**Table S5:** Cell results of C2C12 knock‐down.

**Table S6:** Functional data.

## Data Availability

The code used for differential expression analysis and all bioinformatic plots is available at https://github.com/s‐jessen/b2a_res_single_fiber. The mass spectrometry proteomics data have been deposited to the ProteomeXchange Consortium via the PRIDE partner repository with the dataset identifier PXD064789. All other data supporting the findings of this study are available from the corresponding author upon reasonable request.
