# Supplementary material for: Fibre Type–Specific Proteomics Reveals Shared and Distinct Skeletal Muscle Adaptations to Resistance Training and Beta2‐Adrenergic Agonist
Source: J Cachexia Sarcopenia Muscle. 2026 Jan 25;17(1):e70175. doi: 10.1002/jcsm.70175 (PMC12833500; doi:10.1002/jcsm.70175)
Supplement: Supplementary file 1 — Data S1: Supporting Information. [file JCSM-17-e70175-s008.docx]

**Supplementary Tables**

Table S1: **Protein identifications**

*Tab1:* Before filtering

*Tab2*: After filtering for 70% valid values

Table S2: **Metadata**

Table S3: **Limma results**

*Tab 1:* Baseline differences in fiber type pools (Fig. 2d)

*Tab2:* Within-group changes in RES group; main effect (Fig. 3b)

*Tab3:* Within-group changes in RES group; type I fibers (Fig. 3b)

*Tab4:* Within-group changes in RES group; type II fibers (Fig. 3b)

*Tab5:* Within-group changes in RES group; fiber type x time interaction (Fig. 3b)

*Tab6:* Within-group changes in B2A group; main effect (Fig. 3c)

*Tab7:* Within-group changes in B2A group; type I fibers (Fig. 3c)

*Tab8:* Within-group changes in B2A group; type II fibers (Fig. 3c)

*Tab9:* Within-group changes in B2A group; fiber type x time interaction (Fig. 3c)

*Tab10:* Between-group changes, independent of fiber type (Fig. 5b)

*Tab11:* Between-group changes, independent of fiber type (Fig. 5c)

*Tab12:* Between-group changes, independent of fiber type (Fig. 5d)

Table S4: **Gene set enrichment analyses**

*Tab1:* Within-group results for each fiber type and intervention group (Fig. 4f)

*Tab2:* Two-way interaction; fiber type independent RES vs. B2A (Fig. 5b)

*Tab3:* Two-way interaction; type I fibers RES vs. B2A (Fig. 5c)

*Tab4:* Two-way interaction; type II fibers RES vs. B2A (Fig. 5d)

Table S5: **Cell results of C2C12 knock-down**

Table S6: **Functional data**
