# Supplementary material for: Fibre Type–Specific Proteomics Reveals Shared and Distinct Skeletal Muscle Adaptations to Resistance Training and Beta2‐Adrenergic Agonist
Source: J Cachexia Sarcopenia Muscle. 2026 Jan 25;17(1):e70175. doi: 10.1002/jcsm.70175 (PMC12833500; doi:10.1002/jcsm.70175)
Supplement: Supplementary file 2 — Figure S1: Data integrity and fibre‐type purity. [file JCSM-17-e70175-s001.pdf]

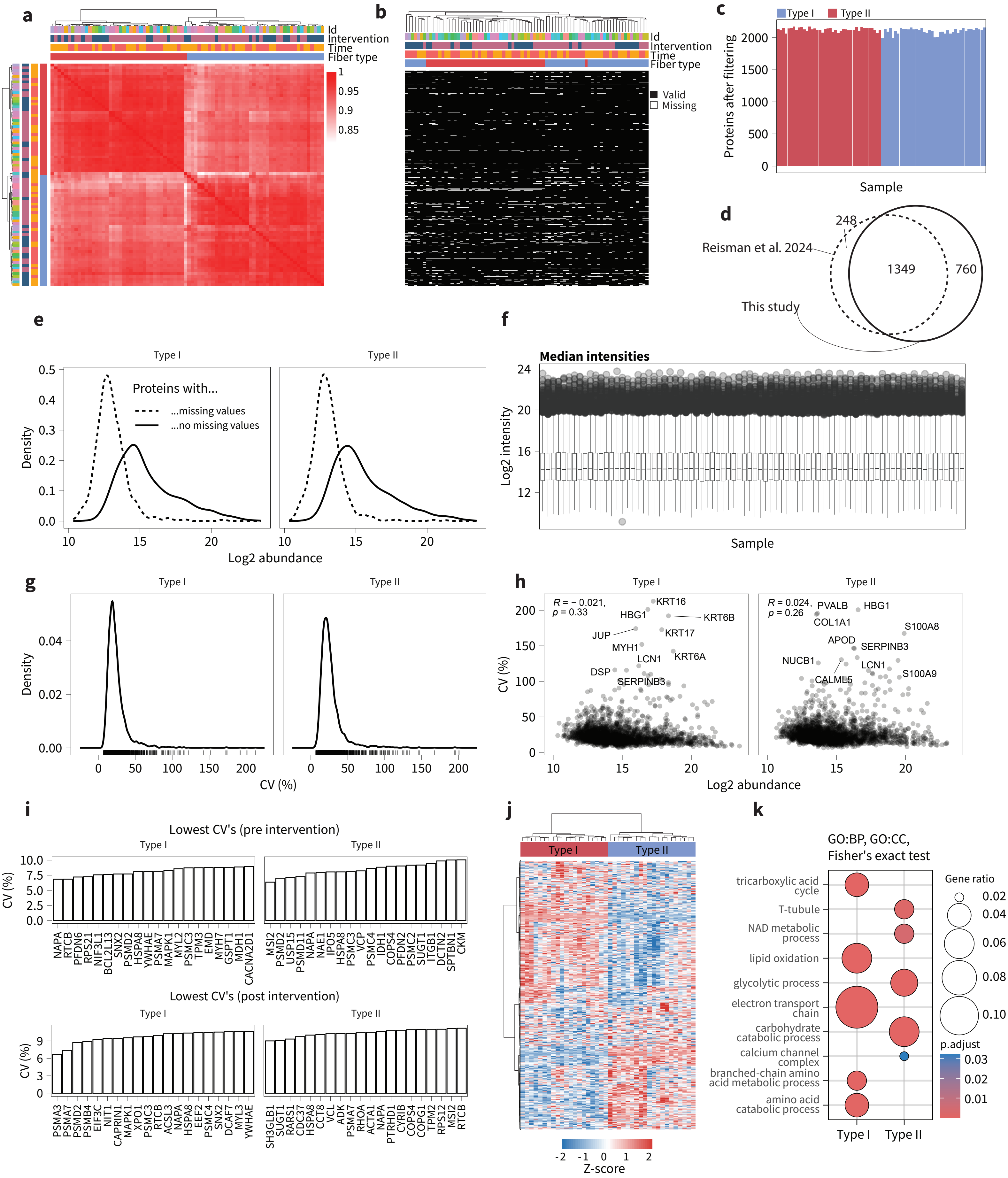

Supplementary Figure 1. Data integrity and fiber type purity. (a), Sample correlations. (b), Missing values after filtering for 70% valid values. (c), Proteins identified per sample after filtering. (d), Comparison of the present study and a recent pooled single-fiber study [46]. (e), Densities of proteins with and without missing values for both fiber types. (f), Median intensity of all proteins for each sample after median scaling. (g), Distribution of correlation coefficient (CV) for both fiber types. CV was calculated as inter-individual, meaning the variability for each protein within all participants. Only pre samples shown in this panel. (h), Relationship between log2 abundance and inter-individual CV for each protein in both fiber types. There was no clear relationship between baseline abundance and CV. (i), The twenty lowest CV’s for each fiber type before (top panels) and after (bottom panels). (j), Heatmap of differentially expressed proteins between type I and type II fibers, using hierarchical clustering (Ward’s) and Euclidean distance. Values are z-scored. (k), Fisher’s exact test on differentially expressed proteins, using GO:BP and GO:CC terms.
